# Aberrant single metastasis to the elbow from primary rectal cancer: a rare presentation

**DOI:** 10.11604/pamj.2020.36.383.21634

**Published:** 2020-08-31

**Authors:** Alessandro Bianchi, Marina Jimenez-Segovia, Jaume Bonnin-Pascual, Marga Gamundí-Cuesta, Myriam Fernandez-Isart, Monica Guillot-Morales, Diego Salinas-Gonzalez, Xavier Francesc Gonzalez-Argenté

**Affiliations:** 1 Department of General Surgery, Hospital Universitario Son Espases, Palma de Mallorca, Spain,; 2 Department of Oncology, Hospital Universitario Son Espases, Palma de Mallorca, Spain,; 3 Department of Traumatology, Hospital Universitario Son Espases, Palma de Mallorca, Spain

**Keywords:** Metastasis, rectal carcinoma, colorectal carcinoma, elbow

## Abstract

Rectal adenocarcinoma usually metastasizes to the liver and lungs and when it has bone spread, it more frequently involves the vertebrae and pelvis. Thus, aberrant metastasis from a rectal adenocarcinoma to upper extremities with preservation of intra-abdominal organs is very uncommon. We present the case of an 80-year-old male patient with a diagnosis of adenocarcinoma of the rectum T4N1M1 with non-axial single bone metastases and with preservation of visceral organs. Anterior resection of rectum after neoadjuvant chemotherapy and radiotherapy were made. The bone metastasis received palliative radiotherapy and was not resected. The patient died 10 months after diagnosis. This clinical situation generally has a poor prognosis. When the patient complains of unusual bone pain it is necessary to suspect a malignant disease and even if extraordinarily rare, rectal cancer must be considered as a possible cause.

## Introduction

Colorectal cancer (CRC) is one of the most common malignancies worldwide. Its usual sites of metastasis are the regional lymph nodes, liver, lungs, breast and prostate. The patient's medical history generally indicates the primary site, and most patients are previously diagnosed in relation to a primary neoplasm and are successively treated accordingly. The most common primary sites are lungs in men and breasts in women [1]. Skeletal metastases are relatively rare in CRC; only 1% of all bone metastases. Osseous metastases are found in less than 25% of cases of CRC, while it is relatively frequent in breast, thyroid, bronchial, renal and prostate cancer [2]. When there is bone involvement, it is generally located at the level of the axial skeleton and the pelvis [3,4]. Rectal cancer has a different lymphatic drainage with respect to colon cancer, leading to the development of more frequent metastatic bone disease. Its proximity to Batson´s venous plexus connects with the vertebral venous system and leads to aberrant metastatic spread [5]. Even though this spread has a primary predilection for the liver and lung, propagation to alternative sites is rare, while spread to bone other than the pelvis or vertebrae is extremely rare [6]. This paper reports a case of a primary single metastasis to the elbow from a rectal carcinoma.

## Patient and observation

**Figure 1:**
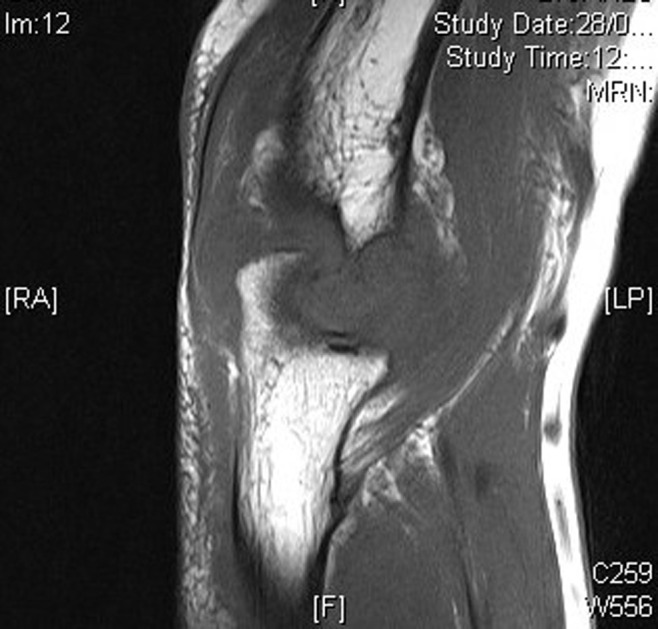
magnetic resonance imaging (MRI) showing the destructive lesion of the bone cortex of the elbow junction

**Figure 2:**
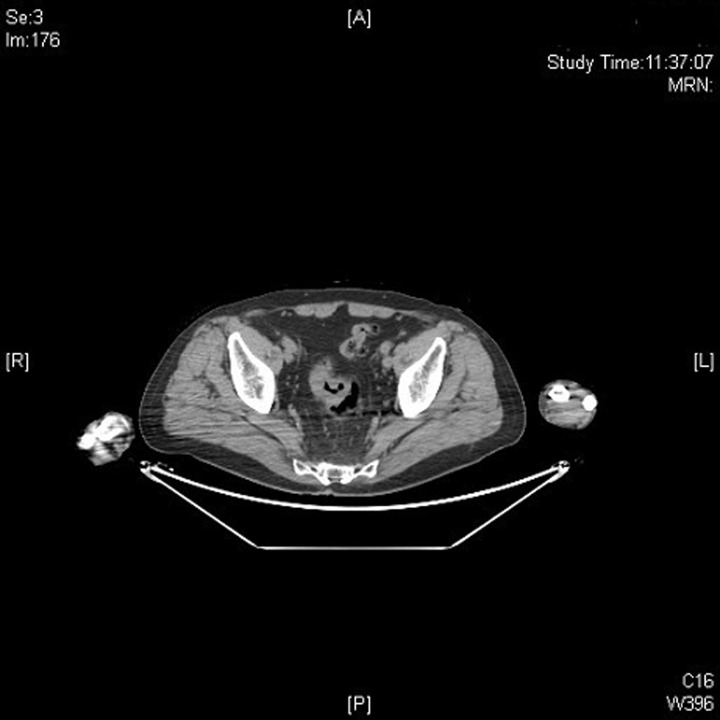
computed tomography (CT) that identifies the primary lesion at the level of the rectum

**Figure 3:**
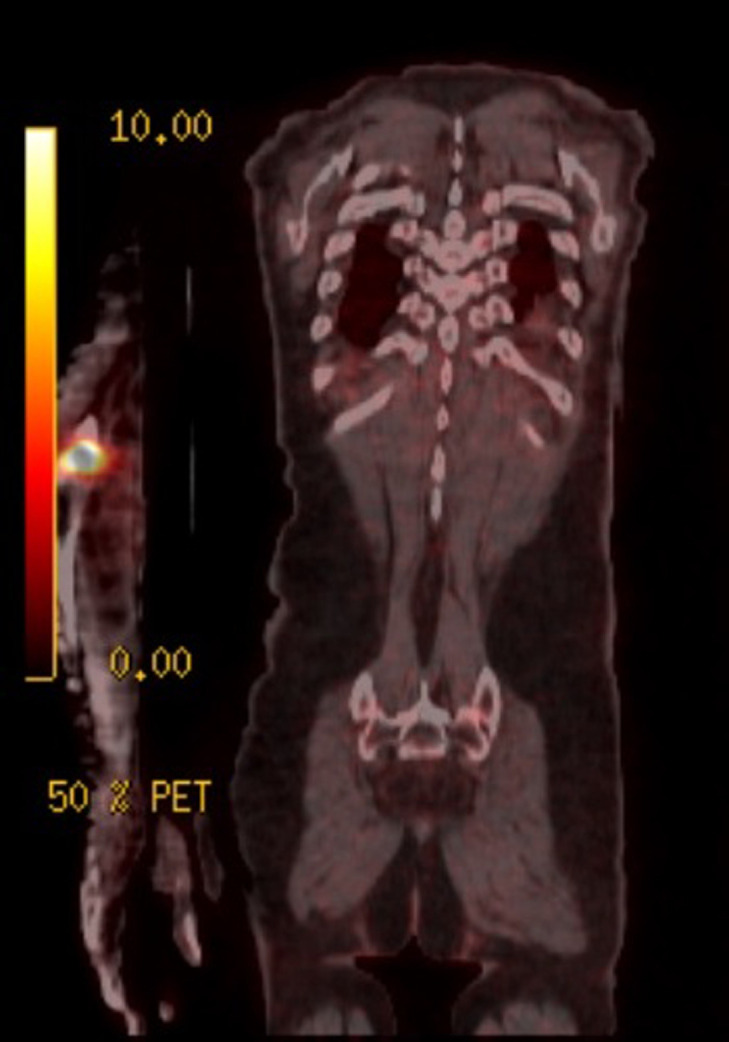
positron emission tomography (PET) that show only the metastatic lesion in the right elbow

We present the case of an 80-year-old male who presented right elbow pain, which was disturbing his sleep and limiting his ability to perform his daily activities. In the anamnesis, 27 years ago the patient was diagnosed and treated for lung squamous cell carcinoma, receiving surgery and postoperative radiotherapy. As an initial part of the study, an elbow x-ray was requested, identifying a bone spur without appreciating any cortex lesion. Due to the persistence and lack of improvement of the symptoms, an magnetic resonance imaging (MRI) was performed, which showed a destructive lesion of the bone cortex of the elbow joint (Figure 1). Computed tomography (CT) scans were performed on the thorax, abdomen, and pelvis to identify the primary lesion and a thickening of the rectum was observed (Figure 2). Bone scintigraphy and positron emission tomography (PET) were performed to rule out distant dissemination of other metastatic lesions. No pulmonary or hepatic metastases were revealed and the only objectifiable metastatic lesion was the right elbow (Figure 3). In direct questioning, the patient reported no occasional bleeding in the rectum or alteration of the bowel movements. The colonoscopy showed a circumferential lesion 9 cm from the anal margin. Biopsy of the rectal lesion and elbow lesion confirmed a poorly differentiated invasive adenocarcinoma of primary and metastatic origin, respectively. The staging of a T4N1M1 lesion was completed with a rectal MRI, which reported rectal lesion extended to perirectal fat with enlarged lymph nodes. Anterior resection of rectum after neoadjuvant chemotherapy and radiotherapy were made. The bone metastasis received palliative radiotherapy and was not resected. The patient died 10 months after diagnosis.

## Discussion

The phenomenon of aberrant skeletal metastasis is relatively rare and the first case was reported in 1870 by Curling *et al*. who described a concomitant lesion in the radius during an autopsy of a patient with rectal cancer [7]. The pathways of metastatic dissemination in rectal cancer are generally multiple. In the first place, dissemination can occur through the peritoneum, generating peritoneal carcinomatosis and ovarian metastases in female patients [8]. Secondly, the tumour cells can spread through lymphatic drainage, affecting the local lymph nodes, paraaortic and even inguinal lymph nodes [9]. Finally, venous dissemination occurs through the middle and lower haemorrhoidal veins that drain the rectum into the inferior vena cava allowing an ascending hematologic spread, reaching the liver, the spleen and ultimately the lungs [10]. Despite this, cases of metastatic dissemination in unusual sites have been described. Cases reported in the literature include the adrenal glands, the pituitary glands and thyroid gland [11], the heart, the bile duct [12], the brain [13] and, as in the case presented, the upper extremities [14]. Bone involvement is not usual for metastatic dissemination of rectal cancer but is typical in other types of cancer such as breast, thyroid, bronchial, renal and prostate cancer [2]. Osseous metastases are found in less than 25% of the cases of CRC. Kanathan *et al*. reported an incidence of skeletal metastases of 1% in a retrospective study of 137 patients with rectal cancer. Due to the lymphatic drainage of the rectum, bone metastases from rectal cancer do not usually appear in peripheral bones, with the typical locations being the vertebrae and pelvis [15]. This could be explained by the presence of Batson's venous plexus, which allows metastases to bypass the portal and cava venous systems. The sacral venous plexus is rich in anastomosis and the distal valvular formation is anatomically variable. The connections between the vertebral and epidural veins associated with a low blood pressure system could cause the blood to pass into the vertebral system [16].

Another peculiar aspect of this clinical case is the bone metastatic dissemination with preservation of intra-abdominal organs. Distal dissemination without organ involvement is an extremely rare event but may result from a combination of host immune response, chemotactic factors of vascular permeability and other mechanisms of cell signalling [17]. Taking into these factors, metastases could spread without following the usual pathways that avoid the venous portal and cava systems and facilitate bone seeding. As in the case presented, the only metastasis detected was located in the right elbow, with both the scintigraphy and PET-CT scan being negative. Patients with colorectal cancer usually have relatively non-specific symptoms: rectal bleeding, bowel movement changes and abdominal pain are the three main symptoms of rectal cancer that may also occur in benign disease. It has been described that many cancer patients usually have more than one symptom [18]. The peculiarity of the case presented is that the patient reported no symptoms or any bowel movement alterations. In a situation of clinical and symptomatic silence, the screening could have detected the appearance of the rectum tumour long before it had become metastatic since the extension study began after the discovery of a lytic lesion of the elbow. The detection of metastasis is generally an unfavourable event, so when an initial diagnosis of metastatic rectal cancer has been made, patients usually have a poor prognosis. Of all the possible metastatic locations described in the literature, the bone location is the worst for average survival, varying between 4 to 7 months according to the series [19]. Bonnheim *et al*. describe an average survival of 10 months when patients suffer a single metastasis and a 6-month survival when bone metastasis is also associated with other distant metastases [20]. In spite of presenting a good tolerance to neoadjuvant therapy and surgery, the patient survived ten months due to progressive degeneration, and probably the persistence of the elbow bone disease.

## Conclusion

We present a case of a patient with primary metastatic rectal adenocarcinoma of the elbow with no evidence of visceral dissemination. This type of pathological entity is particularly rare and, for this, it is essential to maintain a high index of suspicion of an underlying malignancy in unusual cases of bone pain. When aberrant skeletal metastases of rectal adenocarcinoma has been confirmed, the patient is often already at an advanced stage. In general, in these cases the prognosis is poor. This case indicates that careful clinical and pathological evaluations are important in order to make a definitive diagnosis and that palliative treatment should be considered in therapeutic treatment.
